# The relationship between pain intensity and severity and depression in older people: exploratory study

**DOI:** 10.1186/1471-2296-10-54

**Published:** 2009-07-28

**Authors:** Steve Iliffe, Kalpa Kharicha, Claudia Carmaciu, Danielle Harari, Cameron Swift, Gerhard Gillman, Andreas E Stuck

**Affiliations:** 1 Department of Primary Care & Population Sciences, University College London, London, UK; 2 Department of Ageing & Health, St Thomas' Hospital, London, UK; 3 Kings College London, Clinical Age Research Unit, Kings College Hospital, London, UK; 4 Institute of Social & Preventive Medicine, University of Bern, Bern, Switzerland; 5 University Department of Geriatrics, Spital Bern-Ziegler and University of Bern Hospital, Bern, Switzerland

## Abstract

**Background:**

Pain and depression are known to be associated in later life, and both have a negative effect on physical performance both separately and in combination. The nature of the relationships between pain intensity and depression in elderly persons experiencing pain is less clear. The objectives of this study were to explore which factors are associated with depressed mood in older people experiencing pain, and to test the hypothesis that older people experiencing pain are at risk of depressed mood according to the severity or frequency of their pain. In addition we explored whether other potentially modifiable factors might increase the risk of depressed mood in these persons.

**Methods:**

The study is a secondary analysis of baseline data for four hundred and six community-dwelling non-disabled people aged 65 and over registered with three group practices in suburban London who had experienced pain in the past 4 weeks. Intensity and frequency of pain was measured using 24 item Geriatric Pain Measure (GPM) and the presence of depressive symptoms using the 5 item Mental Health Inventory. Risk for social isolation was measured using the 6 item Lubben Social Network scale and instrumental activities of daily living (IADL) were also measured.

**Results:**

Overall 76 (19%) had depressed mood. Pain frequency and severity were not statistically significantly associated with depressed mood in this population. In multivariate analyses, significant predictors of the presence of depressive symptoms were difficulties with basic ADLs (OR 2.8, 95% CI 1.1.7.8), risk for social isolation (OR 4.1, 95% CI 1.8–9.3), and basic education only (OR 2.2, 95% CI 1.1–4.4).

**Conclusion:**

Older people experiencing pain are also likely to experience depression. Among those experiencing pain, social network and functional status seem to be more important predictors of depressive symptoms than the severity of pain. Further studies should evaluate whether improvement of social network and functional status might reduce depressive symptoms in older patients.

## Background

The inter-relatedness of pain and depression can be a clinical challenge resulting in diagnostic and therapeutic uncertainty, and in polypharmacy. A recent survey of nearly 20,000 adults in five European countries found that 28% of those with one depressive symptom had chronic pain, whilst 43% of those with major depression reported chronic pain [1]. The Canadian Community Health Survey of 118 533 community-dwelling adults found that chronic back pain occurred in 20% of clinically depressed adults compared with 9% of the whole population, and that chronic back pain was the strongest predictor of major depression [2]. One view of the relationship between pain and depression is that each can increase the risk of the onset and gravity of the other [3]. An alternative view, supported by empirical evidence from the Baltimore Epidemiological Catchment Area study's data on back pain, is that chronic or repeated episodes of depression doubled the risk of developing pain, but that pain did not increase the risk of depression [4].

Pain and clinical depression are known from other studies to be associated in later life [5], and both have a negative effect on physical performance both separately and in combination [6]. Since the burden of pain-inducing pathologies increases with age, we might expect the relationships between depression and pain to be more obvious in older people. For example, in our community study in London (which had the same study population as reported here) we found that 45% of women and 34% of men aged 65 and over reported pain in the previous four weeks, that pain had a profound impact on activities of daily living, and that there was a significant association between the experience of pain and depressed mood [7]. However, most of those with chronic pain did not have depressed mood, and most of those with depressed mood did not report chronic pain. Other factors must be involved in the relationship between pain and depression, and this paper explores these factors.

According to some studies depressed mood is associated with pain experience in men more than in women [8], and is also directly associated with pain severity [9]. Psychological distress (both anxiety and depression symptoms) in older people experiencing pain may be associated with less education, lower incomes, higher consumption of analgesics, and longer duration of pain [10].

Given these associations, we might anticipate that the risk of depressed mood in older people experiencing pain would be associated with the severity or frequency of their pain, their demographic characteristics, and their functional and cognitive losses. This paper explores these relationships through secondary analysis of data from a community sample of patients aged 65 and over recruited through general practice.

Our primary hypothesis was that amongst older people experiencing pain, depressed mood would be associated with pain frequency and severity. In addition, we hypothesised that depressed mood in those experiencing pain may be associated with advancing age, male sex, being at risk of social isolation, limited education, lower income, functional loss, co-morbidities (as reflected by polypharmacy), and memory loss (which can be a feature of both dementia syndromes and depression).

## Methods

Three large group practices in suburban London were recruited to participate in a multi-centre, multi-national randomised controlled trial investigating the effect of the Health Risk Appraisal for Older persons (HRA-O) instrument on health behaviours and status [11]. The focus of the study was the 'well' old population, and it took place between 2000 and 2003. The HRA-O is a multidimensional, self-completion questionnaire that collects information on health, functional status, health behaviours, preventive care and psychosocial factors in older people. The development of the HRA-O questionnaire, the derivation of its component instruments and the feasibility of its use in British primary care have been reported elsewhere [12,13].

Practices were purposively selected for their interest in primary care for older people, location in suburban London and routine use of electronic medical recording systems in clinical encounters. Local research ethics committee approval was obtained from Brent Local Research Ethics Committee (BEC745) and King's College Hospital Research Ethics Committee (protocol 01–010). A full account of the methodology of the study is available elsewhere [14].

To identify eligible patients aged 65 and over, practice lists were reviewed by general practitioners to identify patients who had moved, died or were ineligible for the study. Eligibility criteria were: those living at home, without A) evidence of need for human assistance in basic activities of daily living, B) high dependency due to major physical or psychiatric illness, or cognitive impairment, or C) a terminal illness. Patients also had to have a sufficient level of English to complete the questionnaires. This patient population was further evaluated using the Probability of Recurrent Admissions (Pra) questionnaire [15] and asked to complete a consent form, by post. The Pra measures the risk of hospital admission and stratifies the population by level of risk for future in-patient care, and was used in the main study as the basis for risk-stratified outcome analyses.

Eligible and consenting patients were posted the HRA-O questionnaire. The HRAO questionnaire contains a number of validated tools, including ones for pain, social network, functional abilities, memory loss and depressed mood. The findings reported in this paper are from the baseline completion of the questionnaire.

Those who gave positive answers to a screening question about the experience of pain in the previous four weeks then completed the Geriatric Pain Measure (GPM), a 24 item multidimensional pain questionnaire that captures information on pain experience (measured on 10 point modified Likert scales) and impact of pain on everyday living [16]. Answers on a scale of 0 to 10 for Pain severity in the last seven days were re-coded to mild (0–3), moderate (4 to 7) and severe (8–10). To identify depressed mood we used the 5 item Mental Health Inventory Screening test [17], a tool validated for screening for depressed mood [18] that does not depend on somatic symptoms, and that categorises respondents as having or not having depressed mood.

Social networks were measured using the six item version [19] of the Lubben Social Network Scale [20], developed specifically for use among older adult populations and used widely in both research and clinical settings [21-24]. On the Lubben scale a scale of nought to thirty captures the extent of social contact with family and friends, and being at risk of social isolation is defined as having a score of less than twelve. Functional ability was assessed using a modified version of the Lawton and Brody activities of daily living scale [25] that captures both basic (BADL) and instrumental activities (IADL). Polypharmacy was defined as receiving four or more prescribed medicines on a regular basis, and changes in walking, climbing steps and getting in or out of cars and buses were measured using the Fried questionnaire [26]. Memory problems were captures using a memory self-report instrument [27]. Information about current income and years of education was collected; those finishing education at 14 or less years were categorised as having basic education only. There was no imputation of missing data for any of these characteristics.

Data analysis was carried out among the sub-sample of elderly persons who answered in the affirmative to a screening question on pain in the last four weeks were included in the dataset. Analyses were carried out using the Statistics Package for Social Sciences (SPSS) [28]. The analyses were conducted in the following sequence:

1) Bivariate analysis using chi square was used to compare those with and without depressed mood for pain frequency and severity, age, sex, living alone, limited education, lower income, functional loss, co-morbidities, and memory loss.

2) Factors associated with depressed mood at p = 0.05 or less were entered in a logistic regression equation with backwards stepwise removal of factors to obtain a parsimonious model. Factors were removed according to their adjusted odds ratio, starting with the least significant associations.

## Results

Four thousand four hundred and sixty six patients aged 65 and over were identified across the three practices, of which 391 were excluded based on *a priori *criteria, so that a total of 4075 older people were sent an invitation letter and a consent form. Overall 2620 of the 4075 returned a completed questionnaire and consent form (64%). Based on the questionnaire 117 were excluded because of self-reported need for human assistance in basic activities of daily living, leaving 2503 people in the study.

After randomisation 1240 older people were posted the health risk appraisal questionnaire at baseline, of which 1090 completed and returned the questionnaire, a response rate of 88%. Those who returned a completed health risk appraisal questionnaire were less likely to describe their health as fair or poor rather than excellent, very good or good, than non-responders (23.1% vs. 34.7% p = 0.002), were on average about one year older than participants, and women tended to refuse more often than men. In other respects responders and non-responders were similar (Stuck et al 2007).

**Figure 1:**
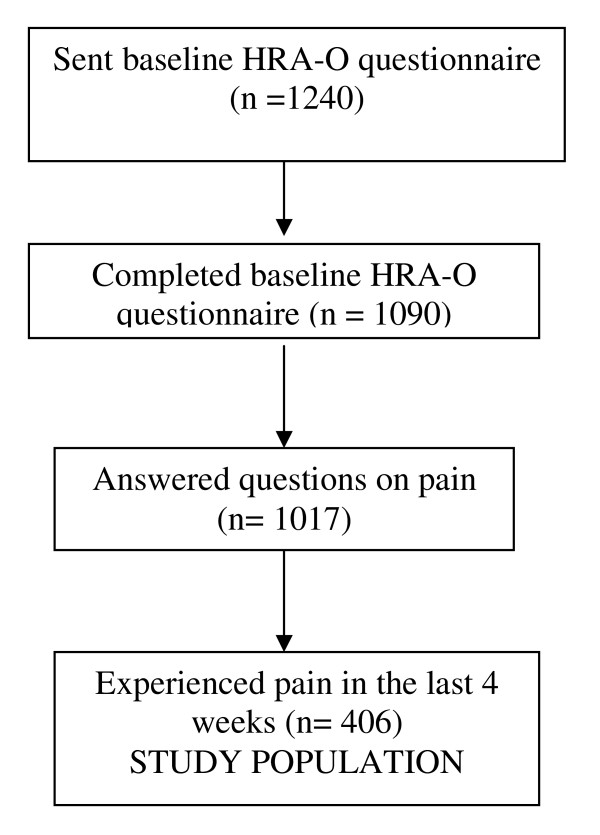
**Derivation of the study population**.

One thousand and seventeen responders (82%) completed the screening question on pain experience in the previous four weeks. Four hundred and six (39.9%) had experienced pain in the past 4 weeks and this group of older people was used for the analysis. Figure 1 shows the derivation of the study sample.

The characteristics of the 406 older people reporting pain in the previous four weeks are shown in table 1. The study population self-reported ethnic origin, and 98% described themselves as white; because of this ethnicity has not been included in the analysis of factors associated with pain and depression.

**Table 1:** Characteristics of older people reporting pain in the previous four weeks (n = 406)

Characteristic	N	%
Age group: 65–74	218	53.7

75–79	101	24.9

80–84	68	16.7

85 and over	19	4.7

Male	157	38.7

Female	249	61.3

Living alone	259	64.3

At risk of social isolation	59	14.6

Depressed mood	76	18.8

Problems with memory	40	10.1

Polypharmacy	179	44.9

Difficulties with BADL	24	6.0

Difficulties with IADL	169	42.9

Recent change in activities	262	67.2

Recent decrease in activities	210	54.3

Basic education only	230	57.1

Receives only state pension	125	31.2

Table 2 shows the factors associated with depressed mood in this sub-population experiencing pain in the last four weeks, both unadjusted and adjusted for other factors. Bivariate analyses suggested that depressed mood was significantly associated with memory problems, taking 4 or more medicines on a regular basis, difficulties with at least one basic and one instrumental activity of daily living, recent reduction in activities, being at risk of social isolation and having had only basic level education. There was no significant association between depressed mood and either the frequency or the severity of pain.

**Table 2:** Factors associated with depressed mood in those experiencing pain

Characteristic	N with depressed mood	% with depressed mood	Unadjusted odds ratio	95% Confidence intervals		p value	Adjusted Odds ratio	95% Confidence intervals		p value
Male	25/155	16.1	1.5	0.9	2.6	0.1	1.5	0.7	2.9	0.3
								
Female	55/243	22.6								

Aged 65–74	41/213	19.2	1.1	0.7	1.8	0.65	0.9	0.5	1.8	0.8
								
Aged 75 & over	39/185	21.1								

Daily pain	42/156	21.2	1.3	0.8	2.2	0.35	1.7	0.8	3.6	0.2
								
Less than daily	30/143	17.3								

Mild pain	34/206	16.5								
								
Moderate pain	36/162	22.2	1.6	1.1	2.3	0.06	1.0	0.55	1.5	0.7
								
Severe pain	9/26	34.6								

Analgesics at least 3 times/week	37/163	22.7	1.3	0.8	2.2	0.3	0.6	0.3	1.3	0.2

Living alone	25/139	18.0	1.0	0.7	1.6	0.6	1.6	0.8	3.3	0.2

Memory problems	16/40	20.5	2.0	1.0	3.9	0.001	2.1	0.8	5.4	0.1

Polypharmacy	42/174	24.1	1.7	1.0	2.7	0.05	1.3	0.6	2.5	0.5

Difficulties with BADL	12/24	50.0	4.4	1.9	10.3	< 0.001	2.8	1.1	7.8	0.05

Difficulties with IADL	44/166	26.5	1.9	1.2	3.1	0.01	1.0	0.5	2.2	0.9

Recent change in activities	59/257	23.0	1.7	0.9	3.0	0.07	1.0	0.4	2.6	0.9

Recent decrease in activities	50/207	24.2	1.8	1.1	3.1	0.025	0.7	0.3	1.7	0.5

At risk of social isolation	22/58	37.9	3.1	1.7	5.6	< 0.0001	4.3	1.8	10.4	0.001

Basic education only	60/227	26.4	3.0	1.7	5.3	< 0.0001	2.2	1.1	4.4	0.03

Receives only state pension	47/270	17.4	0.6	0.4	1.1	0.09	1.5	0.7	3.0	0.30

Unadjusted odds ratios are derived from univariate analyses and adjusted odds ratios are derived from multivariate analyses.

When the significant factors were entered in a stepwise backward regression to obtain the most parsimonious model, three factors remained associated with depressed mood in those experiencing pain. Risk of social isolation and basic level of education remained significant, and difficulty with at least one BADL also had a significant impact on depressed mood. For risk of social isolation the odds ratio was 4.3 (95% CI 1.8 – 10.4, p = 0.001), for basic education only it was 2.2 (95% CI 1.1 – 4.4, p = 0.03) and for difficulties with BADL it was 2.8 (OR, 95% CI 1.1–7.8, p = 0.05),

## Discussion

Depressed mood in older people experiencing pain is not associated with pain frequency or severity but is independently associated with risk of social isolation and having had only basic education. These associations may occur because coping strategies for pain may be less effective for those with weaker social networks or less skill in understanding and managing pain. Attention to social support and pain management skills may be appropriate for clinicians working with older people experiencing pain and associated depressed mood. Difficulty with BADL was also significantly associated with depressed mood, but this finding should be treated with caution because of the small numbers with BADL impairment. Our hypotheses that age, gender, low income, co-morbidity, pain frequency and severity and memory loss would be associated with depressed mood in those experiencing pain were not confirmed.

There are a number of methodological limitations, which should be taken into account when interpreting the results of this study. The sample was drawn from three general practices in suburban London, and subject to eligibility criteria and disability screening implemented for recruitment into a trial of health promotion, which may limit the generalisability of the results. Because of the cross-sectional nature of the study it is not possible to disentangle cause and effect. The prevalence of pain identified within this sample may be lower than that in the general population of older primary care patients, partly because we deliberately excluded disabled older people and partly because the participants were a self-selecting sub-group who returned lengthy questionnaires. The number of older people with severe pain is small, and a type 2 error is possible. The dataset for analysis was constructed around a screening question about pain in the last four weeks, resulting in a wide variety of pain experiences being incorporated into a single group. We also measured depressed mood (which is a common clinical problem in primary care) rather than major depression, which is less common.

### Implications for practice

Daily pain appears to be prevalent among community-dwelling older people and is often untreated [29]. Chronic pain has a wide-ranging impact on daily life and activities [30], including functional limitations, fatigue, sleeping problems, and depressed mood [5] and significantly reduces quality of life [31]. Given the potential impact of pain, clinicians need to be able to accurately assess and understand the nature of pain in everyday life. Pain and depression appear to be closely connected. For example, several studies report that depression is associated with more pain complaints, greater pain intensity, longer duration of pain and greater likelihood of non-recovery [32]. Clinicians working with older people who experience persistent pain and who are also depressed need to consider how best to treat each component of their patient's problem. Given the findings from the literature review [32], one plausible way to alleviate depression in older people experiencing pain may be to treat pain aggressively. However, this simple clinical approach may be complicated by other factors mediating between pain and depression. For example, a study of a large cohort of American women aged 55 to 72 showed that absence of a confidant, having few close friends and few relatives was associated with both higher levels of persistent or recurrent bodily pain and depressed mood [33]. Similarly, persistent depressed mood is associated with significant physical impairment [34]. Those considering treatment of depression in older people experiencing pain may need, therefore, to consider the pain itself, the older person's social relationships and their functional impairments, as well as the depression symptoms.

## Conclusion

Given the limitations of this secondary analysis our conclusions are tentative. In our sample older persons experiencing pain were at risk for depression, as we would predict from the literature. However, contrary to other findings, amongst those experiencing pain social network and functional status seem to be more important predictors of depressive symptoms than the severity of pain. These findings need to be confirmed or refuted in other studies. Further studies should also evaluate whether improvement of social networks or functional status might reduce depressive symptoms as effectively as addressing pain control, in older patients with depressed mood and persistent pain.

## Competing interests

The authors declare that they have no competing interests.

## Authors' contributions

AS, CS, DH & SI conceived, designed and implemented the study on which this paper is based, analysed and interpreted the results and contributed to the writing of this paper; GG and KK managed the study, entered and cleaned all the data, carried out data analysis, interpreted the results and contributed to the writing of this paper; CC undertook the literature review, carried out secondary data analysis and contributed to the writing of this paper. All authors read and approved the final version of the manuscript.

## Pre-publication history

The pre-publication history for this paper can be accessed here:


